# Genome wide prediction of HNF4α functional binding sites by the use of local and global sequence context

**DOI:** 10.1186/gb-2008-9-2-r36

**Published:** 2008-02-21

**Authors:** Alexander E Kel, Monika Niehof, Volker Matys, Rüdiger Zemlin, Jürgen Borlak

**Affiliations:** 1 BIOBASE GmbH, Halchtersche Str., 38304 Wolfenbüttel, Germany; 2 Fraunhofer Institute of Toxicology and Experimental Medicine, Center for Drug Research and Medical Biotechnology, Nikolai-Fuchs-Str., 30625 Hannover, Germany

## Abstract

We report an application of machine learning algorithms that enables prediction of the functional context of transcription factor binding sites in the human genome. We demonstrate that our method allowed *de novo *identification of hepatic nuclear factor (HNF)4α binding sites and significantly improved an overall recognition of faithful HNF4α targets. When applied to published findings, an unprecedented high number of false positives were identified. The technique can be applied to any transcription factor.

## Abstract 2

An application of machine learning algorithms enables prediction of the functional context of transcription factor binding sites in the human genome.

## Background

Regulation of gene expression is accomplished through binding of transcription factors (TFs) to distinct regions of DNA (TF binding sites (TFBSs)), and, after anchoring at these sites, transmission of the regulatory signal to the basal transcription complex. Indeed, regions around TFBSs can be interrogated with regards to binding and interaction with other TFs (so-called composite modules) as well as local sequence properties that favor recruitment of TFs, bending and looping of DNA and nucleosome positioning. Some of these TFs are specific for a particular tissue, a definite stage of development, or a given extracellular signal, but most TFs are involved in gene regulation under a rather wide spectrum of cellular conditions. It is clear by now that combinations of TFs rather than single factors drive gene transcription and define its specificity. Dynamic and function-specific complexes of many different TFs, so-called enhanceosomes [1], are formed at gene promoters and enhancers to drive gene expression in a specific manner. At the DNA level, the blueprints for assembling such variable TF complexes on promoter regions may be seen as specific combinations of TFBSs located in close proximity to each other. They are termed 'composite modules' (CMs) or 'composite regulatory modules' [2] or *cis*-regulatory modules [3]. There may be several different types of CMs located in the regulatory region of one gene, which may be distant from each other (for example, liver- and muscle-specific enhancers of one gene) or overlapping. Taking this into account, it becomes more and more evident that the 'local sequence context' in the vicinity of the TFBS, as well as 'global context' of the whole promoter/enhancer where the TF site is located, influences binding and functioning of the corresponding TF. Numerous examples of so called composite regulatory elements are reported (see the TRANSCompel database [4]) when TF binding and proper functioning of a site is strongly dependent on other sites located in the close vicinity (adjacent or even overlapping sites) or quite distant from each other (up to 100 and more nucleotides). For instance, for the TF family of nuclear receptors (to which hepatocyte nuclear factor (HNF)4 factors belong) there is experimental evidence showing clear dependence between functioning of HNF4 factors at their cognate sites and binding of other factors to the neighboring sites, both synergistically and antagonistically [4]. There is a need to develop computational models to predict TFBSs that are functional and are involved in the control of gene transcription. Recent developments in the field of machine learning techniques allow us to apply them to build highly sensitive and specific methods for predicting functional TFBSs in human and other genomes.

Because of our continued interest in the regulation of liver-enriched TFs [5,6], we were particularly interested in identifying novel genes regulated by the hepatic nuclear factor (HNF)4α. Indeed, HNF4α is a versatile TF, and several investigators have reported the identification of genes targeted by HNF4α. These studies included various experimental approaches, including transient transfection of HNF4α into a human hepatoma cell line, a rat insulinoma cell line, and a human kidney cell line [7-9]. Additionally, findings with conditional knock-outs of HNF4α [10] were recently reported. Notably, in the study of Odom *et al*. the genome-wide identification of binding sites for TFs HNF4α, HNF1α, and HNF6 was reported by use of the ChIP-chip assay with a 13,000 human promoter sequence containing microarray [11]. Strikingly, in the case of HNF4α, the number of contacted promoters was unexpectedly high; 1,575 potential HNF4α target genes were identified. In addition, 42% of the genes occupied by RNA polymerase II were also occupied by HNF4α, suggesting that nearly 50% of all liver-expressed genes are regulated by HNF4α alone. Similarly, in another recent ChIP-chip experiment of ENCODE (Encyclopedia of DNA Elements) genomic regions (about 1% of the human genome), 663 novel HNF4α binding sites were identified in 100 genes [12], which suggests there are a large number of HNF4α targets (over 60,000 sites in the vicinity of about 10,000 genes) if extrapolated to the entire genome. This unprecedented high number of HNF4α binding sites revealed by the ChIP-chip method raises the question of the functional role of all these sites in the regulation of gene transcription.

Indeed, the ChIP-chip assay is a much wanted and a highly advanced method for the genome-wide search and identification of TFBSs. Nonetheless, it suffers from unacceptably high false positive findings. In the study of Odom *et al*. [11] 252 (16%) false positive binding sites were predicted by the authors. Another problem with this method is that a surprisingly small fraction of identified ChIP fragments possesses the canonical binding motif for the corresponding TF [3]. This limitation needs to be overcome, and it is highly desirable to identify functional binding sites relevant for the regulation of gene transcription. Furthermore, in the current studies there is often no rationale for the selection of promoters spotted on the array; for example, no bioinformatic approach is applied to identify relevant sequences for the design of the ChIP-chip assay.

Here, we report a computational approach based on a novel machine learning technique, which enabled the identification of genome-wide TFBSs. This method was applied to search for HNF4α gene targets. A genetic algorithm and an exhaustive feature selection algorithm were trained on 73 known and well characterized HNF4α target sequences in promoters and enhancers of different mammalian genes (Additional data file 1). By genome-wide scanning of all human gene promoters we identified novel genes targeted by HNF4α. Then, a subset of predicted binding sites was confirmed by electromobility shift assay. We further interrogated promoter sequences for HNF4α binding sites identified by the ChIP-chip assay. We also analyzed expression of genes targeted by HNF4α and observed a good correlation between computationally annotated HNF4α binding sites and expression of targeted genes. Notably, ChIP-chip experiments tend to report a rather high number of TFBSs in promoters of genes whose regulation by HNF4α is not observed, whereas our computational method for the prediction of HNF4 regulatory sites enabled improved specificity with the method encompassing rules for the regulation of gene expression.

Overall, we demonstrate the power of our computational approach in identifying novel genes targeted by HNF4α. Our machine learning technique significantly improved the overall recognition and, therefore, the identification of faithful HNF4α targets. This method enabled refinement of TF site predictions based on the ChIP-chip assay and identification from among them of potentially functional sites, as reported here. Furthermore, our method can easily be applied to the genome-wide identification of genes targeted by any mammalian TF and is not limited to promoter sequences alone, with an overall success of approximately 80% based on experimental confirmation.

## Results

### Repeats in HNF4 binding sites

**Figure 1:**
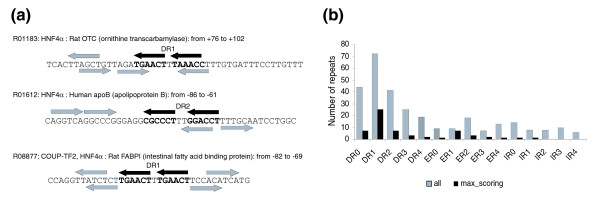
Repeats in the structure of HNF4 binding sites (from TRANSFAC^®^). **(a) **Examples of multiple repeats forming canonical DR1 as well as DR2, inverted (IR) and 'everted' (ER) repeats. The centrally located black arrows, marked as DR1 or DR2, indicate the repeat with the maximal score (sum of the scores of single elements) as compared to the gray arrows representing multiple repeats. **(b) **Statistics of repeats of different types (direct repeats, DR0-4; everted repeats, ER0-4; inverted repeats, IR0-4) in the structure of HNF4 sites. Black bars show the observed number of repeats found in the structure of 73 sequences of known HNF4 binding sites (listed in Additional data file 1) considering one repeat with the maximal score per sequence. Gray bars show the total number of repeats found in this set of HNF4 sites.

It is generally accepted that HNF4 regulates gene expression by binding to direct repeat motifs of the RG(G/T)TCA sequence separated by one nucleotide (direct repeat (DR)1) [13]. We used two 'half-site' positional weight matrices (PWMs) taken from the TRANSFAC^® ^database (see Materials and methods) in order to identify such repeats in the sequences containing known binding sites of HNF4 (based on TRANSFAC^® ^annotation; Additional data file 1). We found that although the DR1 repeat structure is clearly seen in the general consensus and in the full positional weight matrix, actual genomic sites often can be characterized by more complicated structures. The results are presented in Figure 1. As can be shown, the current common point of view that DR1 is the only characteristic repeat for HNF4 binding sites is not accurate. We can identify repeats at various distances and with various orientations different from the canonical DR1 structure in the sequences experimentally known as true HNF4 binding sites. This fairly unbiased analysis of the internal repeat structure of known HNF4α binding sites confirms earlier observations that sometimes HNF4α binds to elements other then DR1.

### Molecular organization of the local context of genomic HNF4α binding sites

**Figure 2:**
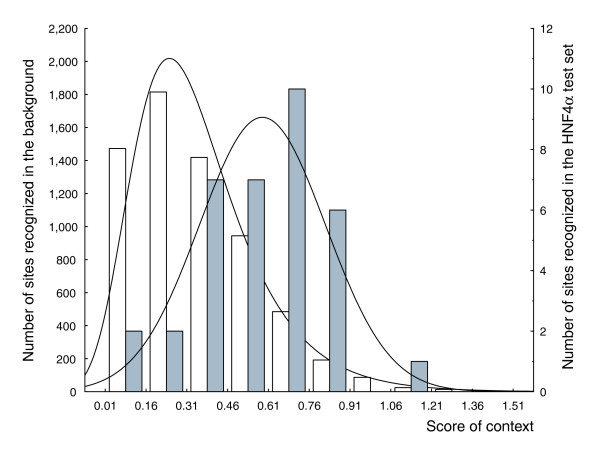
Two histograms showing the distributions of the score of context in the -28 bp/+33 bp flanks of real HNF4α sites (gray bars, see also test set; Additional data file 1) versus the -28 bp/+33 bp flanks of PWM matches (PWM score >0.8) in random genomic positions (white bars). Mean values of the two distributions are 0.5849 and 0.348, respectively. The x-axis gives the score of context; the left y-axis gives the number of PWM matches in random genomic positions with the corresponding score of context; and the right y-axis gives the number of real HNF4α sites with the corresponding score of context.

We applied the 'local context' machine learning technique to the set of known HNF4α binding sites in order to reveal properties of the DNA context in close proximity to the functional HNF4α binding sites. We analyzed frequencies of short oligonucleotides of length 4, as well as the frequency of short repeating motifs of lengths 2 and 4. The binding sites for HNF4α are characterized by various repeat structures (Figure 1a). From our analysis of distribution of half-site motifs above we can see that short additional degenerated motifs resembling parts of the consensus repeat can be seen in the vicinity of the core of the site. Based on these results we decided to perform a thorough contextual analysis of DNA sequences containing HNF4α binding sites. The analysis was done by applying the algorithm in an exhaustive search through the space of all possible short oligonucleotides and repeats in various regions of the sites and their flanks. In addition, we searched for non-redundant sets of contextual features as reported previously [14]. Table 1 presents the results of this analysis. We selected a combination of four oligonucleotides, six dinucleotide pairs and six four-nucleotide repeats that are overrepresented or underrepresented in the sequences of genomic HNF4α binding sites and compared the results to background sequences. A linear combination of these local contextual features gives rise to the score of context (*d *in equation 1; see Materials and methods). Figure 2 depicts two distributions of the score of context that we obtained on a test set of HNF4α recognition sites (the test and training sets are defined in Additional data file 1) and the test background set. Splitting the set of sites into the training and test subsets was done by random selection. Note that the sites from the test set were not used in the training phase of the algorithm. As shown in Figure 2, we clearly discriminate real HNF4α sites from false positives in the background. In our further analysis, we used the score of context with a cut-off value of 0.55, which minimizes the sum of false negative error (the proportion of unrecognized real sites to the total number of HNF4α sites in the test set) and false positive error (the proportion of false recognition of the background sequences as true sites to the total number of tested sequences in the test background set).

**Table 1:** Oligonucleotides and short repeats found in the local context of genomic HNF4α sites

Oligonucleotide/repeat	Mode	Wind_from	Wind_to	*r*_*min*_	*r*_*max*_	Alpha	Avrfreq_Y	Avrfreq_N
MDDR	(I)	22	66	0	0	0.003082	13.433 (3.665)	11.051 (4.782)
ANGB	(I)	20	38	0	0	0.016132	5.358 (2.529)	3.582 (2.845)
CDDM	(I)	36	38	0	0	0.020372	4.346 (2.332)	3.212 (1.732)
AV-VS	(II)	1	34	33	33	0.008246	6.694 (3.663)	4.893 (3.088)
MD-DB	(II)	20	70	20	25	-0.003212	16.941 (3.078)	14.711 (3.344)
BR-NT	(II)	33	37	9	18	-0.003942	4.802 (5.460)	7.702 (5.144)
VS-YA	(II)	1	34	11	11	0.0103	4.237 (1.742)	3.121 (2.640)
VB-HA	(II)	1	34	5	5	0.008647	9.028 (2.985)	6.517 (3.741)
HM-GN	(II)	40	50	2	4	0.006468	7.783 (4.335)	4.672 (3.764)
(RBNH)^2^	(III)	20	51	5	12	0.030376	7.259 (1.778)	5.961 (2.168)
(MVKN)^2^	(III)	20	51	7	13	0.015979	3.123 (1.413)	2.388 (1.155)
(BNDK)^2^	(III)	32	32	7	7	-0.002214	0.000 (0.000)	14.343 (28.652)
(DNCD)^2^	(III)	28	42	7	7	0.068635	4.176 (2.797)	1.051 (2.196)
(NBHV)^2^	(III)	26	26	7	7	-0.001045	0.000 (0.000)	13.626 (28.102)
(NVYB)^2^	(III)	29	29	7	7	-0.001696	0.000 (0.100000)	12.909 (27.523)

Mode: (I), search for oligonucleotides; (II), dinucleotide pairs; (III), four-nucleotide repeats. Wind_from and Wind_to, sequence window in which the motif was found (the HNF4α site is located between positions 29 and 42, flanks are 28 bp and 33 bp long, respectively). *r*_*min *_and *r*_*max*_, distances between dinucleotide pairs and repeats. Alpha, coefficients in the linear function. Avrfreq_Y, the average frequency of the oligonucleotides in the corresponding window among sequences of the real sites. Avrfreq_N, background sequences. The standard error is given in parentheses.

Among selected contextual features, some, like the motifs ANGB and MDDR, fit to different parts of the HNF4 consensus sequence and appear to be overrepresented in a rather wide area around the center of the binding sites (Table 1). The motif CDDM is overrepresented in quite a small area corresponding to the central positions of the sites. Very interesting are the 'negative' features, such as repeats of the motif BNDK, which are positioned at the beginning and the end of the HNF4 consensus, and repeats NBHV and NVYB, which have one part of the repeat just at the left edge of the consensus and the second part located at the center of the consensus. Such 'negative' features represent some nucleotide combinations that are rarely or never observed at functional binding sites, although such sequence context can be found in the background sequences. It is important to mention that the background sequences were generated as matching the HNF4 PWM but still have the additional contextual differences that can be found through the local context approach. Therefore, the local context approach can capture contextual rules that cannot be identified by the conventional PWMs, since they distinguish real sites from false positive hits of the matrix.

To validate the contextual features found in our analysis, we ran the algorithm 3 times using different samples of 100 background sequences generated in the same way as the first sample. As expected (see Materials and methods), the resulting set of identified contextual features was different each time (data not shown), whereas, the oligonucleotides ANGB and CDDM, as well as the repeat (RBNH)^2^, were identified in all tested cases (although with slightly different 'from' and 'to' parameters of the sequence window). Overall discrimination of the test distributions using the obtained sets of contextual features was practically the same as obtained in the first run shown in Figure 2. Therefore, in all further analyses we used the set of features obtained in the first run.

### Molecular organization of the global context of HNF4α binding sites

To study the global context, we retrieved flanking sequences of length ±500 bp around known HNF4 binding sites (Additional data file 1) and put them into the Y_Global _set. The background set (N_Global_) was constructed based on randomly chosen intergenic fragments of DNA from various human chromosomes applying the same strategy as for N_Local_ (described in Materials and methods), but with the 500 bp flanks around the assumed false positive match of the HNF4 matrix (we chose at random 642 sequence fragments scattered through intergenic regions on all human chromosomes).

**Figure 3:**
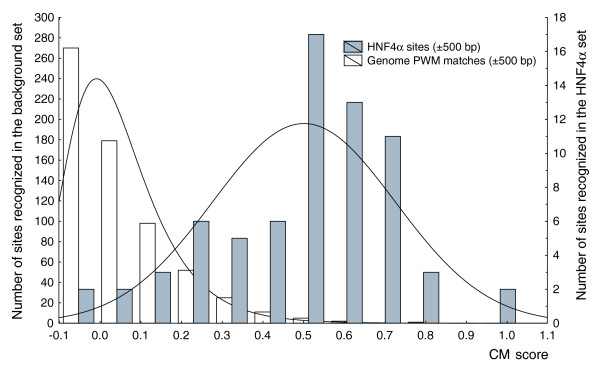
Two histograms showing the distributions of the CM score in the ±500 bp flanks of HNF4α sites (gray bars) versus ±500 bp flanks of PWM matches (PWM score >0.8) in random genomic positions (white bars). The average CM score for real HNF4α sites is 0.499, whereas for PWM matches in random genomic positions (in the set N_Global_) it is 0.050 (ratio = 9.98, *t*-test *p*-value = 1.4896 × 10^-26^).

We analyzed these sets using the Composite Module Analyst (CMA) program (see Materials and methods), which allowed us to study combinations of TFBSs in the interrogated sequences. Input for CMA is a set of DNA sequences under study (foreground set) - for example, the set of HNF4 functional sites - and a set of background sequences. By comparison of two sequence sets, CMA identifies through an iterative genetic algorithm a specific combination of TF matrices (PWMs) that are common for the foreground set of sequences and distinguish them from the background sequences [15]. The results are given in Table 2. The CMA algorithm identified six single TF matrices and eight pairs of TF matrices characterized by variable distances between sites in each pair (*d*_*max *_is defined as a distance of 100, 200 and 500 bp). Figure 3 shows the results of the comparison of the distributions of the CM score in the two sets: the Y_Global _set (HNF4α sites ±500 bp; gray bars) and the N_Global _set (Genome PWM matches ±500 bp in random genomic positions; white bars). One can see the clear discrimination between these sets. The average CM score for real HNF4 sites equals 0.499, whereas for random genome PWM matches it equals 0.050 (ratio = 9.98, *t*-test *p-*value = 1.4896 × 10^-26^).

**Table 2:** Matrices and matrix pairs of the global context selected by the CMA program

Matrix_ID(1)	TFs(1)	Cut-off(1)	Matrix_ID(2)	TFs(2)	Cut-off(2)	*d*_*min *_(bp)	*d*_*max *_(bp)	*κ*	*φ*
**V$MAZ_Q6**	**MAZ**	0.89						4	0.020763
**V$ER_Q6**	**ER-α**	0.913						4	0.047177
**V$HEB_Q6**	**HTF4**	0.969						4	0.078905
V$HNF4_Q6_01*	**HNF4α, HNF4α2, HNF4γ**	0.976						4	0.210340
**V$HEN1_02**	**HEN1**	0.854						4	0.099368
V$CREB_Q2*	**CRE-BP2, CREM, ATF-1,2,3,4,6**	0.888						4	0.086618
V$HNF4_Q6_01*	**HNF4α, HNF4α2, HNF4γ**	0.8325	**V$EFC_Q6**	**RFX1 (EF-C)**	0.6825	8	100	2	0.043344
**V$COUP_01**	**COUP-TF1, COUP-TF2**	0.8005	**V$KROX_Q6**	**Egr-1,2,3,4**	0.8315	8	100	2	0.053285
**V$PEBP_Q6**	**PEBP2α/AML1,3; PEBP2β**	0.84	**V$TEL2_Q6**	**Tel-2a,b,c**	0.878	8	100	2	0.214469
**V$ELK1_01**	**Elk-1**	0.785	**V$WHN_B**	**FOXN1**	0.948	8	100	2	0.111909
**V$CMYB_01**	**c-Myb, B-Myb**	0.86	**V$KROX_Q6**	**Egr-1,2,3,4**	0.841	8	100	2	0.100922
V$FOXO1_02*	**FOXO1,2,4, FOXJ3**	0.8715	**V$FXR_Q3**	**FXRα/RXR**	0.8135	8	500	2	0.100184
V$HNF4_Q6_01*	**HNF4α, HNF4α2, HNF4γ**	0.8065	**V$HNF4_01**^(*)^	**HNF4α, HNF4α2, HNF4γ**	0.8705	8	200	2	0.080381
**V$XBP1_01**	**XBP-1**	0.8845	**V$FOXO1_02**^(*)^	**FOXO1,2,4, FOXJ3**	0.8715	8	200	2	0.112402

Matrix_ID(1) and Matrix_ID(2) are the TRANSFAC^® ^identifiers of the selected single matrix (or the first matrix in a pair and the second matrix in the pair, respectively). The other headings of the table correspond to the parameters of the composite module score (see equation 2 in Materials and methods). The first six lines of the table represent the single matrices selected by the algorithm to represent the global context; the other lines represent the pairs of matrices selected by the CMA program. The corresponding values of the parameters (in the Cut-off, *κ *and *φ *columns) are optimized by the CMA algorithm. *Matrices corresponding to TFs whose binding site combinatorial co-occupancy was found in Odom *et al*. [45] for promoters of liver-expressed genes. Bold text indicates TFs identified by the CMA.

The obtained significant combination of matrices determines the global context that is characteristic for the regulatory regions around functional HNF4α binding sites in the genome. The biological interpretation of located composite modules is based on the concept of the 'enhanceosome', postulating that, for a proper performance of regulatory function, a TF, while binding to the DNA target sites, should participate in many protein-protein interactions with other TFs binding in the neighborhood of the sites. As can be demonstrated, the algorithm selected HNF4 matrices three times, for example, as a single element, as well as parts of matrix pairs with another HNF4 matrix and with the V$EFC matrix. Note that the algorithm additionally selected TF matrices corresponding to recognition motifs of, for instance, MAZ, ER, FOX, CREB, Elk1 (Ets domain factor), COUP-TF, RFX1 and some others. Strikingly, it is known that HNF4α TFs cooperate with ER [16] and build synergistic composite elements with CREB [17,18] and antagonistic composite elements with COUP-TF [19] (see also the TRANSCompel^® ^entries C00369, C00129, and C00124). Interaction and cooperation between some other factors listed in the composite module is also known, for example, COUP-TF with ER [20] and CREB with Ets [21]. Thus, the found composition around known HNF4α binding sites represents potential interaction partners of HNF4 factors, therefore providing functionality in the regulation of HNF4α target genes. Note that in the case of computing the global context there was no test set available, that is, all known sites were used to train the algorithm. In order to validate the computed composite module, we performed a series of ten data shuffling experiments. Each time, the assignments of positive and negative sets were randomly shuffled among the sequences and CMA was applied in order to find a matrix combination that would best discriminate between these sets of sequences. No good discrimination was obtained in such shuffling iterations. The maximum ratio achieved between the mean values was 1.6 with *t*-test *p-*values of 10^-5^, which is much higher than in the unshuffled case (Figure 3).

### Complex criteria for determining functional HNF4α binding sites

We determined the following complex recognition criteria for a sequence of length 1,000 bp to be a potential target for HNF4α TFs: the maximal matrix score of an HNF4 site in the sequence (*q*_*max*_) should be >0.8; the maximal local context score (*d*_*max*_) should be >0.28; the maximal global context score (CM;* v*_*max*_) should be >0.18; the sum of matrix scores of all HNF4 sites found in the sequence (*q*_*Sum*_) should be >10.0; and the TFBS with the maximal score should be considered as the binding site for HNF4α, whereas the 1,000 bp regions provide the functional context for this site.

These rather complex criteria were derived through an iterative computation of different combinations of each individual threshold with the goal of achieving a method that would have approximately 90% sensitivity and would efficiently use individual criteria of the local and global contexts. Finally, we obtained criteria that yield 87% sensitivity on the Y_Global _set (known functional sites for HNF4 factors with 500 bp flanks) and thresholds of the local and global context scores were set at the minimum of the sum of errors of these two criteria (Figures 2 and 3, respectively). As can be seen from these two figures, the relative contribution of the global context to prediction power is larger than that of the local context. The sum of the errors for the local context is approximately two times higher than the sum of the errors for the global context. This means that in applying these complex criteria, in approximately 13% of cases we may miss an identification of functional HNF4α binding sites (the false negative rate of the method is 13%).

### Analyzing ChIP-chip data for HNF4α sites

**Figure 4:**
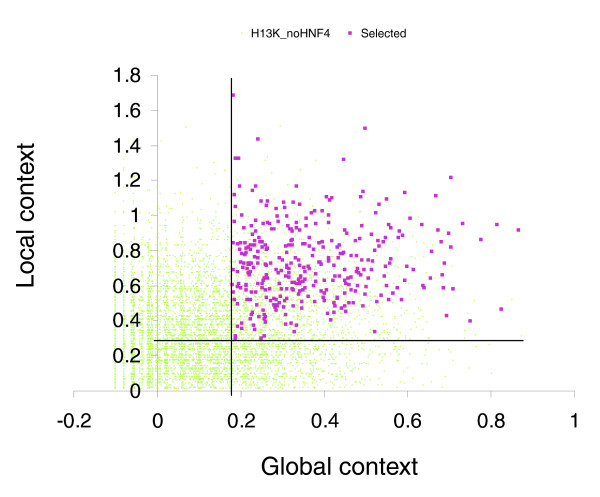
Plot of the distribution of global and local contexts in the 375 sequences (red squares) selected from the 'positive' set of ChIP-chip results reported by Odom *et al*. [11] versus all 10,852 sequences from the 'negative' (not binding; H13K_noHNF4) set (green dots) reported for the same experiment. The selected sequences are characterized by the highest global and local context scores whereas the majority of the 'negative' sequences are characterized by low values for these two scores. The vertical and horizontal lines show two thresholds chosen for the global context score (0.28) and the local context score (0.18).

**Figure 5:**
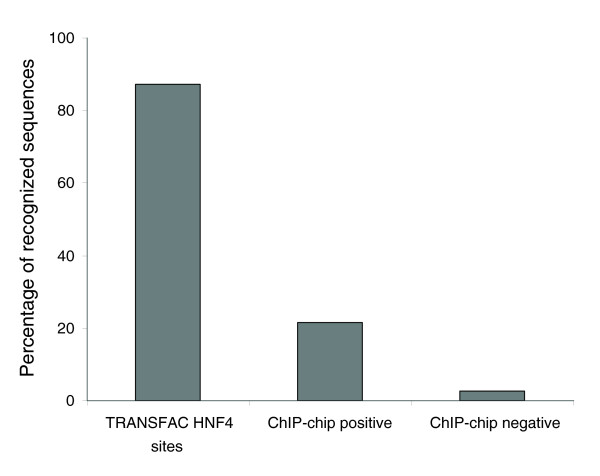
Percentages of sequences passing the complex recognition criteria in the set of known HNF4 binding sites (TRANSFAC^® ^HNF4 sites), in the set of 'positive' sequences based on ChIP-chip experiments reported by Odom *et al*. [11] for hepatocytes and in the set of 'negative' sequences described by Odom *et al*. [11]. From the last set we estimate that the percentage of false results from our method is about 2.6%.

Using the HNF4α PWM, which was built on a representative set of 73 known functional HNF4α binding sites in mammalian genes, and two new methods (local and global content for estimating the DNA context around functional HNF4α binding sites as discussed above), we analyzed the ChIP-chip data for HNF4α reported by Odom *et al*. [11]. We interrogated two sets of sequences: 'positives', a set of 1,605 sequences that were reported as HNF4α-targeted genes in hepatocytes; and 'negatives', a set of 10,852 sequences that were reported not to be contacted by HNF4α in hepatocytes and pancreatic islets. The average length of the sequences reported by Odom *et al*. was approximately 1 Kb [11]. In each sequence of both sets, we computed the number of potential HNF4α binding sites (matrix score >0.8), the sum of the scores for all sites, and the maximal score of the sites found in the sequence. Thereafter, we calculated the local context score (*d*) and the global context score (*v*) for each potential HNF4α binding site in these sequences and reported the maximal scores obtained in each sequence. We applied the complex recognition criteria (see above) to the sequences in these two sets. As a result, only 21% of the 'positive' set (that is, 375 sequences out of 1,605) passed the criteria. Indeed, 79% of the sequences were rejected, since they did not pass one or several requirements as defined above. In order to estimate the rate of false positives of our method, we applied it to the set of 'negative' sequences. Our complex criteria rejected 97.4% of these sequences, giving us an overall estimate of 2.6% for the false positive rate. Figure 4 depicts a plot of the global and local context scores, comparing distribution of the 375 sequences selected from the 'positive' set versus distribution of all sequences in the 'negative' set. Obviously, the selected sequences are characterized by the highest global and local context scores, whereas the majority of the 'negative' sequences are characterized by low values for these two scores. The list of the 375 sequences that passed our criteria are given in Additional data file 2. Furthermore, Figure 5 summarizes the data obtained in the analysis of known HNF4α binding sites, as well as 'positive' and ' negative' sets of sequences derived from ChIP-chip experiments reported by Odom *et al*. [11]. These data clearly show that the majority of the sequences revealed in the ChIP-chip experiments of Odom *et al*. [11] differ quite significantly in their local and global context from the sequences of known and experimentally confirmed HNF4α binding sites. We estimate that only 20% of these sequences fulfill our requirements to be considered as faithful functional HNF4α binding sites. Note that Odom *et al*. [11] assume a 16% false discovery rate in the identification of binding sites in their ChIP-chip experiments. Application of our analysis to the Odom *et al*. data suggests about 80% of the ChIP-chip identified targets do not meet the contextual requirements that characterize biologically functional sites and, therefore, may not be involved in HNF4α-dependent regulation of gene transcription.

### Linking HNF4α binding sites to gene expression

We also applied our computational method to data reported by Naiki *et al*. [7] and Lucas *et al*. [9]. Notably, these investigators carried out microarray experiments to identify genes whose expression differed upon targeted overexpression of HNF4α. From these studies a list of differentially expressed genes was obtained. Additionally, we compared the differentially expressed genes with findings reported by Odom *et al*. [11], who performed ChIP-chip experiments with HNF4α. We thus compared data from two different approaches, that is, targeted overexpression of HNF4α and ChIP-chip data for the identification of novel HNF4α target genes. We then applied our computational approach (by use of the complex recognition criteria described above) to interrogate the data sets. The results are presented in Table 3. Only a small fraction of identified genes could be compared directly; 75 and 70 differentially expressed genes (Up + Dn) reported by Naiki *et al*. [7] and Lucas *et al*. [9], respectively, and 150 genes whose expression did not change (NC). As can be seen from the data given in Table 3, our computational method and the ChIP-chip data are similar when correlated with the gene expression data of HNF4α-targeted genes (see the Table 2 legend); approximately 18-20% of differentially expressed genes were similarly identified by the ChIP-chip and our computational method based on the data of 145 differently expressed genes. Indeed, several genes targeted by HNF4α were identified by both methods (for example, 5 genes (*ACADVL*, *RBKS*, *SLC35D1*, *ATP7B*, *MGST2*) out of 70 from the Lucas *et al*. [9] data set).

**Table 3:** Comparison of gene lists between HNF4α expression data, ChIP-chip data, and computational prediction of target promoters

	Gene sets in ChIP-chip experiment		HNF4 targets identified by PWM V$HNF4_Q6_1 (cut-off = 0.9)	HNF4 targets identified by local + global context
			
	Positive*	Negative^†^		
Gupta *et al*. [44] Up + Dn (133)	13 (9.8%)	ND	66 (49.6%)	41 (30.8%)
Naiki *et al*. [7] Up + Dn (75)^‡^	17 (22.7%)	32 (42.7%)	15 (20%)	14 (18.7%)
Lucas *et al*. [9] Up + Dn (70)^‡^	13 (18.6%)	39 (55.7%)	17 (24.3%)	13 (18.6%)
Lucas *et al*. [9] NC (150)^‡^	20 (13.3%)	99 (66%)	29 (19.3%)	4 (2.7%)

*The number of differentially expressed genes with HNF4α binding sites as identified by ChIP-chip experiments. ^†^The number of differentially expressed genes with no HNF4α binding as determined by ChIP-chip experiments. ^‡^The number of genes whose expression was upregulated or downregulated by more than two-fold. NC, genes with no change of expression; ND, not determined.

At the same time, our computational method for identifying HNF4α gene targets is less error-prone; there were 2.7% false results based on the computational method compared to 13.3% false results determined using the ChIP-chip method (based solely on gene expression data from Lucas *et al*. [9]) (Table 3, last row). It is of considerable importance that when using just a single HNF4 PWM and ignoring local and global sequence context the prediction of HNF4α target genes becomes prone to generating false positive errors (19% as shown in Table 3).

### Search for HNF4α functional sites amongst all known human gene promoters

We applied the method developed for identifying putative HNF4α gene targets to the full set of promoters of human genes annotated in TRANSPro™ database release 2.1 (containing 15,455 promoters). First, we scanned promoters in the region from -500 to +100 around the transcription start site (TSS) for matches of the HNF4 weight matrix with matrix score *q *> 0.8 accompanied by local context score *d *> 0.48. We identified 3,009 promoters that had at least one site passing both these criteria. Next, we chose the highest scoring match of the HNF4 matrix in each of the promoters and retrieved 500 bp flanking regions around the match. We applied the complex criteria (see above) to obtain a set of sequences, which led to the prediction of 375 target promoters; among them 121 promoters of genes encoding TFs and other components of the cell signaling system. These genes attracted our attention for experimental verification by electrophoretic mobility shift assay (EMSA) as reported here. The full list of the predicted target promoters is given in Additional data file 2.

### Electrophoretic mobility shift assay confirmation

**Figure 6:**
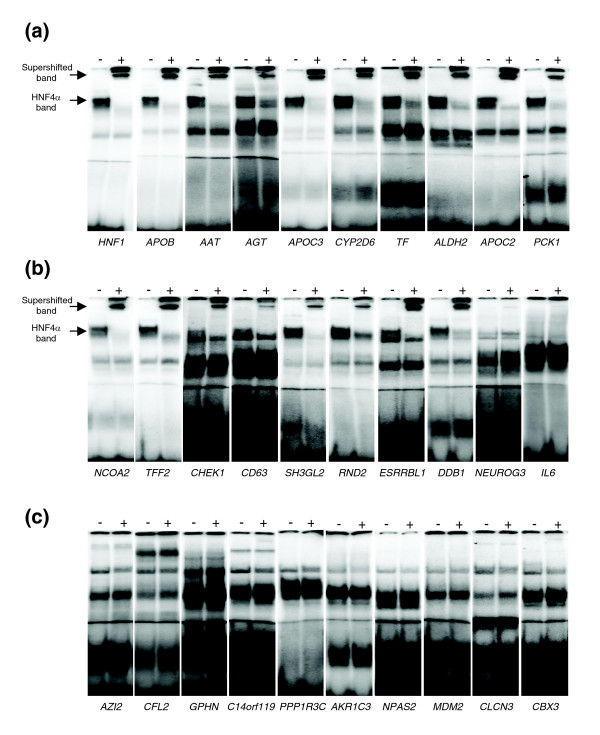
EMSA confirmation experiments. **(a) **EMSA with established HNF4α recognition sites. Electrophoretic mobility shift experiment with 2.5 μg Caco-2 cell nuclear extracts and oligonucleotides corresponding to promoter regions derived from *HNF1*, *APOB*, *AAT*, *AGT*, *APOC3*, *CYP2D6*, *TF*, *ALDH2*, *APOC2 *and *PCK1 *as ^32^P labeled probes. For supershift analysis an antibody directed against HNF4α was added (+). **(b) **EMSA with predicted novel HNF4α recognition sites. Electrophoretic mobility shift experiment with 2.5 μg Caco-2 cell nuclear extracts and oligonucleotides corresponding to promoter regions derived from *NCOA2*, *TFF2*, *CHEK1*, *CD63*, *SH3GL2*, *RND2*, *ESRRBL1*, *DDB1*, *NEUROG3 *and *IL6 *as ^32^P labeled probes. For supershift analysis an antibody directed against HNF4α was added (+). **(c) **EMSA with potential recognition sites from putative HNF4α targets reported by Odom *et al*. [11]. Electrophoretic mobility shift experiment with 2.5 μg Caco-2 cell nuclear extracts and oligonucleotides corresponding to promoter regions derived from *AZI2*, *CFL2*, *GPHN*, *C14orf119*, *PPP1R3C*, *AKR1C3*, *NPAS2*, *MDM2*, *CLCN3 *and *CBX3 *as ^32^P labeled probes. For supershift analysis an antibody directed against HNF4α was added (+).

Supershift experiments with probes for established recognition sites for HNF4α, that is, promoter regions derived from HNF1α, AAT, APOB, AGT, APOC3, CYP2D6, TF, ALDH2, APOC2 and PCK1, resulted in binding of HNF4α (Figure 6a). This exemplifies the selectivity and sensitivity of the EMSA assay for validating HNF4α binding sites for ten arbitrarily chosen but known targets of HNF4α. From the list of 375 predicted HNF4α target genes (see above) we selected a further 10 novel HNF4α binding sites for experimental confirmation that are characterized by high PWM and local and global context scores and that were not reported in the study of Odom *et al*. [11]. Note that EMSA revealed binding of HNF4α to NCOA2, TFF2, CHEK1, CD63, SH3Gl2, RND2, ESRRBL2 and DDB1, whereas supershift experiments did not confirm HNF4α binding to NEUROG3 and IL6 (Figure 6b), thus providing an estimate of 80% for the sensitivity of our computational method for *de novo *prediction of HNF4α binding sites. A summary of the biological function of these newly identified HNF4α target genes is given in Table 4.

**Table 4:** Biological functions of novel predicted HNF4α gene targets

Gene symbol	Gene name	Biological function
*CD63*	*CD63 antigen (melanoma 1 antigen)*	Localization plasma membrane
		Endocytosis
*CHEK1*	*CHK1 checkpoint homolog*	Cell cycle
		Negative regulation of cell proliferation
		DNA damage response
*ESRRBL1*	*estrogen-related receptor beta like 1*	Induction of neuronal apoptosis
*DDB1*	*damage-specific DNA binding protein 1, 127 kDa*	DNA repair
*NCOA2*	*nuclear receptor coactivator 2*	Regulation of transcription
		Signal transduction
*RND2*	*Rho family GTPase 2*	Signal transduction
		Protein transport
		Dendrite development
*SH3GL2*	*SH3-domain GRB2-like 2*	Central nervous system development
		Signal transduction
		Endocytosis
*TFF2*	*trefoil factor 2*	Defense response
		Digestion

In addition, we wished to verify HNF4α binding sites predicted by ChIP-chip experiments [11]. Note that nearly 80% of the proposed HNF4α binding sites were rejected by our computational method, which combines analysis of HNF4 matrices with the local and global contexts of the sequences. For this we selected ten genes that were reported by Odom *et al*. [11] to be targeted by HNF4α in hepatocytes but were characterized by our computational method with extremely low scores for the HNF4 weight matrix and local and global contexts (all four tests comprising the complex criteria set by us failed to identify these genes as HNF4α targets). Therefore, these ten potential sites (in promoters of genes *NPAS2*, *GPHN*, *PPP1R3C*, *AKR1C3*, *CFL2*, *MDM2*, *CLCN3*, *CBX3*, *AZI2 *and *C14orf119*) were analyzed for HNF4α binding. Strikingly, none of these sites were bound by HNF4α, as shown by the supershift experiments (Figure 6c).

## Discussion

*De novo *computational identification of genes targeted by various TFs is a challenging task, especially in genomes of higher eukaryotic organisms, which are characterized by extremely large gene regulatory regions. Indeed, binding of TFs to their cognate sites on DNA is a complex process that requires the presence of a specific short sequence pattern in DNA, commonly described by a PWM. Furthermore, the specific local sequence context in the vicinity of the binding site is required to provide favorable conditions for DNA confirmation and DNA flexibility [22]. In addition, local structures such as short repeats and palindromes are often observed and, as discussed before, are needed to enable an optimal environment for homo- and heterodimerization of TFs [4]. The particularly important role of the global context of TFBSs in determining cooperative binding of factors with other TFs to their neighboring DNA sites is broadly recognized [1]. A broad collection of experimentally proven facts on cooperative binding of two and more TFs to so-called composite regulatory elements with synergistic effects on the regulation of gene expression is provided by the TRANSCompel^® ^database [4]. Among these are several known examples of nuclear receptors that are involved in such composite elements (for example, glucocorticoid receptor, androgen receptor and others). But there are no bioinformatics tools available so far that enable a systematic analysis of the combinatorial sequence context of genomic binding sites.

In general, there is a definitive need to develop novel computational approaches to improve the description of the DNA patterns required for TF binding. Ellrott and co-workers [23] applied a Markov chain model to identify HNF4α binding sites in order to improve recognition accuracy of the DNA binding pattern. They have demonstrated that the approach performs better than PWMs alone, but this approach does not consider any local context on the flanks of sites that indeed play a crucial role in promoter activation and DNA binding *in vivo*.

Recently, local context in the form of short repeats has been successfully implemented to improve recognition of binding sites for nuclear receptors [24,25]. Extending the previously published approach [24] to the application of hidden Markov models, Sandelin and Wasserman [25] modeled various known constellations of direct, inverted and everted repeats for different sites of nuclear receptors and were able to improve the general precision of the recognition. This approach looks very promising, although it lacks any capability to classify predicted sites in order to identify which particular TF from the large family of nuclear receptors is able to bind to the predicted sites. In addition, we show here that binding sites for such nuclear receptors as HNF4α are highly enriched by various different repeat structures, which does not completely fit with the existing paradigm that the DR1 repeat comprises the canonical structure of HNF4 sites. This makes it extremely difficult to judge factor recognition based on an oversimplified model based on the repeat structures of sites.

We therefore developed a novel approach for the recognition of functional HNF4α binding sites by analyzing the local and global contexts of targeted genes. The method is based on the assumption that the sequence context surrounding TFBSs in DNA is very important for the process of TF binding to the site and, most importantly, for providing specificity of the TF in the regulation of gene expression - by either activation or repression of the gene in particular cellular situations. The sequence contexts of the TFBSs actually makes them functional: in the absence of the proper context, the possible binding of a TF to a particular site on DNA can be impaired or made functionally neutral (which means that the factors are bound to the DNA, but do not influence expression of the gene; such sites are, therefore, non-functional).

In the current work, we performed a thorough analysis of the local nucleotide context on the flanks of known functional HNF4α sites, as well as in the whole local region occupied by the sites. We improved our earlier published approach to analyzing local context [2], which is based on a SiteVideo method [14], and introduced new types of contextual features that modeled various repeated structures in the sequences on the flanks of the sites. Interestingly, the revealed short oligonucleotide features and repeats can be classified into three categories. The first category includes oligonucleotides like ANGD and MDDR and repeats like AV-VS, VS-YA and (RBNH)^2 ^that fit to different parts of the HNF4 consensus sequence and appear to be overrepresented in a rather wide area around the center of the binding sites. We can interpret such features as a signature of overrepresentation of HNF4 site-like patterns in the local area surrounding the functional HNF4 site, which may play a role in increasing the probability of HNF4α binding to this site. The second category includes oligonucleotides like CDDM and repeats like (DNCD)^2^, which are overrepresented in a quite small area corresponding to the central positions of the sites. Such features correspond to the central HNF4 site pattern, but they reveal some contextual features of the functional HNF4 sites that cannot be described by the PWM matrix model, for example, correlation between neighboring nucleotides that can not be captured in full by the mononucleotide weight matrix. The third category includes 'negative' features that reveal oligonucleotides to be underrepresented at functional binding sites when compared with background sequences. Such negative features can be local, as in the case of the repeats (BNDK)^2^, (NBHV)^2 ^and (NVYB)^2^, which again describes some mutual nucleotide correlations that cannot be captured by PWMs, or distributed, such as BR-NT, which can be interpreted as an 'echo' of some physical-chemical properties of DNA that may interfere with the binding or functioning of the TFs. Notably, the length of the oligonucleotides tested by our method was restricted to four letters of the extended code, mainly because of the high computational complexity of the calculations; however, this oligoncleotide length seems quite optimal for revealing statistically significant features of DNA sequences.

We assume that, in addition to the local context, the global context of the TFBSs in the regulatory regions of genes dictates whether these sites are functional. The global context, which we model by specific combinations of binding sites of various TFs, provides some sort of 'scaffold' on DNA to enable cooperative or antagonistic interactions between TFs. These multiple and complex interactions, if correctly organized in space and time, give rise to the regulatory function of the TFBSs under investigation. It is clear by now that binding of a single TF to its cognate site on DNA alone does not guarantee the proper functional activity of the targeted gene. More interaction with other TFs in the transcription complex and in the enhanceosome are necessary to acquire the full regulatory functionality.

Specifically, known functional combinations of TFBSs were used before in a number of promoter analysis approaches, for example, for identifying muscle-specific promoters [26,27], the promoters of liver-enriched genes [28], yeast genes [29], immune-specific genes [30-32], and the promoters of genes regulated during the cell cycle [33] or genes involved in antibacterial defense responses [34,35]. A number of approaches identifying composite motifs were created, including BioProspector [36], Co-Bind [37], MITRA [38], and dyad search [39]. These programs help to discover *ab initio *new regulatory sites for yet unknown TFs. Another set of methods has been developed to discover composite modules by utilizing information on potential binding sites for known TFs (stochastic methods such as ClusterScan [40] and TOUCAN system [41], and probabilistic methods such as reported in [42]). We combined these two approaches by computing local context as an exhaustive *ab initio *composite motif discovery method with the global context - the powerful composite module discovery method based on application of a genetic algorithm.

Furthermore, we wish to point out that our method considers several alternative PWMs for the calculation of the global context. The use of such alternative PWMs for constructing the composite module (see Materials and methods) enables more reliable predictions. In cases with small training sets but with data derived from multiple rounds of computations, the use of different matrices is particularly meaningful. These computations are far from statistical saturation, but new sites may eventually add a certain bias and potentially drive the new PWM matrix away from the functional binding site sequence context. In the case of HNF4α we included both half-site and full-site matrices. Still, the full-length PWMs are able to capture some subtle differences in the spacing sequences between the 'repeats'. Recent reports confirm our old observation that such 'gap' sequences between two repeats of the nuclear receptor sites are often actually more conserved between species then the repeats themselves. Therefore, the combined usage of fixed-length PWMs together with the distributed oligonucleotides on the flanks provides a more robust method for site detection than each method separately.

To compare the findings from our algorithm with the best existing methods, we performed an independent run of the TOUCAN software [41] on the set of HNF4α sites. Notably, TOUCAN is similar to our method and is based on a genetic algorithm. It identified a combination of 14 PWMs for different TFs, including two matrices for HNF4α factors (V$HNF4_01 and V$HNF4_01_B) and matrices for other factors (V$USF_01, V$OCT1_02, V$SP1_Q6, V$PPARG_03 and some others). Interestingly, except for the HNF4 matrices, there were no further correlations with matrices selected by our method (Table 2). This difference can be explained by the ability of our method to identify pairs of matrices and also by the ability to optimize the cut-off values, which is not possible in TOUCAN. Another advantage of our method is the possibility to include information on the tissue specificity of the factors, through extensive use of factor expression annotation in the TRANSFAC^® ^database. We further compared our method with the NUBIscan algorithm [24]. Our approach combines many different features of the most conserved part of the sites as well as various features of the local and global contexts, whereas the NUBIscan algorithm relies solely on the 'repeated' structure of the nuclear receptor sites, which is indeed a very profound property of these sites but not the only one. And, similar to the later published Wasserman approach [25], the NUBIscan algorithm lacks the capability to classify predicted sites in order to identify which particular TF from the large family of nuclear receptors is able to bind to a predicted site. Notably, our method was designed specifically for the recognition of HNF4 sites. Nonetheless, our strategy to define the local and global sequence contexts is a highly generalized method and can be applied to any TF.

An additional point of consideration is the 'regulatory potential score', as introduced in the work of Elnitski and co-workers [43], which refers to the five-way multi-species alignment introduced in the UCSC Genome Browser. In our study we did not restrict ourselves to conserved sites based only on multispecies homology of regulatory regions (phylogenetic footprinting). Although this concept is quite popular for the selection of evolutionarily conserved regulatory sites, the method suffers from low sensitivity because functional TFBSs are frequently missed, mainly due to the very complicated evolutionary history of mammalian regulatory sequences, which can hardly be modeled by the simple divergent concept, which is the basic concept of phylogenetic footprinting.

Furthermore, promoter regions are characterized by very specific average base composition as well as composition of di-nucleotides. It is known that whereas the overall genomic sequences are highly depleted of CG dinucleotides, promoters are often located near high concentrations of them, in or near so-called CpG islands. We therefore compared the nucleotide and dinucleotide composition of the promoter sequences of the known HNF4α sites and sequences that were used as probes in the ChIP-chip experiments of Odom *et al*. [11], since these were the sequences to which our method was applied. We did not find any significant difference in the nucleotide and dinucleotide compositions of these two sets of sequences, which is not a surprise since the probes of the chip were designed using known genomic promoters. Consequently, since training and test sequences are very similar in their context, the CpG bias, if any, of the HNF4-containing promoters of the training set cannot bias the results of the analysis of the sequences from the ChIP-chip experiment.

Additionally, many authors attribute a certain functional role to the CpG islands in promoters. These islands can be some sort of centers of regulated DNA methylation, which can effectively contribute to hepatocyte-specific gene regulation by providing HNF4α binding sites with necessary functional context. The absence of such a CpG context in the vicinity of HNF4α binding sites may potentially render them functionally neutral. Therefore, some CG dinucleotide-like features that were included in the local context (for example, elevated frequency of the oligonucleotide CDDM, where D is (A/T/G) and M is (A/C)) may reflect this 'CpG' bias of functionally active promoter sequences and, therefore, help to identify the functionally active HNF4α binding sites.

We further studied the influence of the distance from HNF4α sites to TSSs. As shown in Additional data file 1, the location of known HNF4 sites is variable in promoter sequences and may range from a position close to the TSS to up to +10 kb and -11 kb. Under such high variability, it is improbable that the method can 'memorize' the position of TSSs during training since sequences in the training set are not aligned in relation to TSSs.

We then applied the developed methods to analysis of the data derived from ChIP-chip experiments reported by Odom *et al*. [11] for HNF4α. This study is based on chromatin immunoprecipitation combined with DNA-DNA hybridization on a microarray containing 13,000 human promoter sequences. In the study of Odom *et al*. [11], the number of HNF4α-targeted promoters was unexpectedly high; 1,575 potential HNF4α target genes in hepatocytes were identified, corresponding to 42% of the genes occupied by RNA polymerase II. Only 48 (3%) of the 1,575 putative HNF4α targets were verified, however, in separate gene-specific ChIP-chip experiments. Additionally, HNF4α DNA binding was not distinguished from protein-protein interactions, as *in vitro *binding was not analyzed. We applied our algorithm to the proposed HNF4α gene targets and found merely 20% of them to obey the complex computational criteria (the presence of appropriate local and global contexts) that can predict the functional activity of these binding sites. We further stratified our approach by comparing HNF4α functional sites identified by us with independent gene expression data. This comparison shows that our computational approach is versatile and predicts expressed genes directly targeted by the HNF4α TF with similar sensitivity to chromatin immunoprecipitation (ChIP)-chip experiments. In strong contrast, the false discovery rate of the computational method is almost five times lower than that of the ChIP-chip method. This confirms our suspicion that many of the HNF4α binding sites predicted by Odom *et al*. [11] are functionally neutral, whereas the developed computational method is able to recognize the functionally active HNF4α binding sites based on verification of the local and global contexts of these sites.

Furthermore, in a recent paper by Gupta *et al*. [44], regulation of gene expression was studied in pancreatic cells of an HNF4α conditional knockout model. Expression analysis identified 133 genes as HNF4α regulated. Regulated genes could be compared with the promoter array data of Odom *et al*. [11]. Surprisingly, the overlap between differentially expressed genes and those bound by HNF4α is rather small. In other words, of 133 genes whose expression was dependent on HNF4α, only 13 have been identified by the location analysis reported by Odom *et al*. [11]. Likewise, of 587 promoters occupied by HNF4α in the study of Odom *et al*. [11], 574 showed no significant change in gene expression [44]. Therefore, 86% of HNF4α-targeted genes proposed by Odom *et al*. [11] did not differ in gene expression in the absence of HNF4α. These estimates agree well with our computational approach where only 20% of the target genes proposed by Odom *et al*. [11] could be computationally confirmed.

In the most recent study [45] by the same investigators and through the application of an improved ChIP-chip assay, more then 4,000 HNF4α target genes were identified. By comparing the list of genes identified in the ChIP-chip assay with the list of genes expressed in liver, the authors determined the combinatorial co-occupancy of binding sites of different factors in promoters of HNF4α target genes. Furthermore, this feature correlated well with expression of these genes in hepatocytes. This agrees well with our findings and confirms the utility of our method in defining the local and global contexts for specific combinations of different TFBSs in the vicinity of functionally active HNF4α binding sites of promoters of genes whose expression is regulated by HNF4α. Notably, the combination of PWMs identified by the genetic algorithm (Table 2) captured two TFs, FOX and CREB. Strikingly, these factors were identified independently by Odom *et al*. [45] in an analysis of TFBSs that co-accrued with HNF4α sites (Table 2, matrices indicated by asterisks).

In a further study of Odom *et al*. [46], the authors showed that two-thirds of the binding sites identified by ChIP-chip experiments are not conserved between human and mouse. Taking into account the quite conservative liver expression patterns of genes between these two species, we can conclude that by far not all HNF4α binding sites identified by the ChIP-chip method directly contribute to the regulation of gene expression.

To experimentally validate our predictions, we selected two sets of promoters. The first set contained ten *ab initio*, and therefore novel, HNF4α recognition sites predicted by the computational complex recognition criteria described above. Strikingly, eight of the ten binding sites (*NCOA2*, *TFF2*, *CHEK1*, *CD63*, *SH3Gl2*, *RND2*, *ESRRBL2 *and *DDB1*) could be confirmed as HNF4α binding sites in electromobility supershift experiments (Figure 6b). In addition, we studied another set of ten promoters that were reported by Odom *et al*. [11] as targets for HNF4α, but our computational method rejected them because of extremely low scores for the HNF4 weight matrix as well as low scores for local and global contexts. None of these sites (*NPAS2*, *GPHN*, *PPP1R3C*, *AKR1C3*, *CFL2*, *MDM2*, *CLCN3*, *CBX3*, *AZI2 *and *C14orf119*) did in fact bind to HNF4α, as shown by electromobility supershift assays (Figure 6c). These findings suggest a high error rate concerning the proposed targets by Odom *et al*. [11].

Finally, another computational approach has been applied to analyze the same set of HNF4α (as well as HNF1 and HNF6) ChIP-chip data that is the focus of our current study. Indeed, Smith and colleagues [47] demonstrated that an application of combinations of motifs allowed for improvements in the prediction of the genomic location of TFBSs. In contrast to our approach, however, they performed a blind motif discovery instead of using the existing TF weight matrices. To the best of our knowledge, this makes the algorithm very complicated and increases the risk of missing important TF combinations that are characteristic of functionally active regulatory sites.

Several further improvements to our algorithm can be considered in the future. Among the most important, we should consider the possibility of taking into account sequence conservation in the non-coding regulatory regions of genes between different species, for example, human and mouse. It was demonstrated in recent studies [48-51] that sequence conservation can be a good indication of the functional importance of a region. Indeed, such regions can bear functional TFBSs. Despite being quite useful, such considerations should be taken with care since regulatory regions are characterized by a high level of convergent evolution, which can provide non-divergent means of forming the functional context of a TFBS.

Another direction for further improvements is considering known protein-protein interaction data between different TFs. Such data are partially available in databases such as TRANSFAC^®^, TRANSPATH^® ^and BIND. Known interactions between TFs can help to find proper combinations of neighboring binding sites for these factors.

A further step in improving our method will be the use of PWMs for factors whose expression is tissue specific, as indicated in TRANSFAC^®^. This will greatly improve the predictive power of the method. To achieve this, more extensive annotation of expression information of TFs is needed and will be a task of the future. One possibility for obtaining this information resides in ChIP-chip experiments in conjunction with gene expression data. This will help to identify TF activity in a given cellular environment.

Taking gene expression data into account will significantly help to determine the global and local sequence contexts and, therefore, functional TFBSs. Recently, we applied the algorithm described in this paper to analysis of promoters of differentially expressed genes [2,15,51]. Such an integrative approach is now available in the software system ExPlain™ for a mechanistic interpretation of gene expression changes in eukaryotic cells under various physiological and pathological conditions [52].

## Conclusion

We report a new approach based on machine learning techniques for the *de novo *identification of novel HNF4α binding sites. The genetic algorithms developed by us significantly improved data analysis of various experimental sources. The method described here can be applied to any TF and enables computational prediction of genome-wide functional TFBSs. By applying our method, interactions between different TFs can be taken into account. This provides clues to the mechanisms responsible for promoter activation and even for antagonistic binding of TFs, for example, HNF4α and Coup-TF, which successfully compete for the same binding site but differ in activity under various biological conditions. Indeed, while both factors can bind to the same sequence, the individual local and global sequence contexts determine the actual binding activity and may, therefore, provide an estimate of TF activity in particular cellular or physiological conditions.

## Materials and methods

### Databases

Databases provided by BIOBASE GmbH were used, for example, TRANSFAC^®^, which is a database on gene regulation [53]. It collects data on TFs and their binding sites in promoters and enhancers of eukaryotic genes as well as a library of PWMs. This work was done with TRANSFAC^® ^release 9.4. Additionally, to retrieve promoters of human genes, we used TRANSPro™ release 2.1 [54], which is based on genomic sequence from Ensembl release v35, November 2005. Final verification of the composite modules was done with the help of the TRANSCompel™ database [4].

### HNF4α binding sites in the human genome

In this work, we significantly updated the collection of known genomic HNF4α sites in TRANSFAC^®^. Additional data file 1 lists the collected sites with information about the target genes, positions in the promoters of the genes, and the site sequence.

First of all, we compiled all known HNF4 binding sites from the literature and extended them upstream (28 bp) and downstream (34 bp); this is set as Y_Local_. Next, we prepared the background sequences; this is set as N_Local_. After that, we split the Y_Local _set into two parts: the training set and the test set (sites included in the training set are indicated in Additional data file 1 by asterisks). We also split the N_Local _set into two parts: the training background set and the test background set. The training of the method was done by comparison of training set versus training background set. The testing of the method and building of the histogram in the Figure 2 was done on the test set versus the test background set - on two sets that were not used in the training. This procedure of preparing four sets is the best possible statistical procedure for training and testing of the recognition methods.

### Positional weight matrix for HNF4α binding sites

Based on the collection of HNF4α sites we constructed a PWM (accession number M01031) and two 'half-site' matrices (accession numbers M01032 and M01033) and deposited them in the TRANSFAC^® ^database (Table 5). The construction of PWMs was done according to the general outline described in [51] and as detailed in the protocol of TRANSFAC^® ^matrix construction (see the TRANSFAC^® ^documentation). The half-site matrices were created by manual splitting of each site into two parts and were used independently for the alignment. Together with pre-existing HNF4α matrices in TRANSFAC^® ^(accession numbers M00762, M00764, and M00967), the new matrices were used to search for HNF4α binding sites in genomic sequences. For this basic search we employed the MATCH™ algorithm, calculating scores for the matches by applying the so-called information vector [55]. This algorithm is implemented in the ExPlain™ software system. This software was also used for analysis of the flanking regions of HNF4α sites to search for other TFBSs from the most up-to-date library of matrices derived from the TRANSFAC^® ^Professional database. The cut-offs for the matrices were set to minFN to maximize the sensitivity of the site prediction (false negative rate of 10%).

**Table 5:** Positional weight matrix for HNF4α sites (TRANSFAC^® ^accession number M01031, identifier V$HNF4_Q6_01)

A	**19**	**5**	**11**	**5**	**2**	**52**	46	**49**	**0**	**3**	**3**	**1**	**46**	17
C	**8**	**2**	**3**	**16**	**48**	**1**	2	**0**	**0**	**1**	**19**	**47**	**2**	15
G	**21**	**46**	**30**	**20**	**2**	**0**	10	**9**	**58**	**26**	**7**	**2**	**6**	14
T	**10**	**5**	**14**	**17**	**6**	**5**	0	**0**	**0**	**28**	**29**	**8**	**4**	12
Consensus*	**N**	**G**	**G**	**N**	**C**	**A**	A	**A**	**G**	**K**	**Y**	**C**	**A**	N

*Two 'half-matrices' (M01032, V$HNF4_Q6_02; and M01033, V$HNF4_Q6_03) corresponding to the DR1 repeat are shown in bold and refer to the frequency of a nucleotide of the matrix.

### Machine learning techniques for building methods of identification of genomic sites

To identify novel and functional HNF4α binding sites in the human genome, we first analyzed flanking regions of DNA binding sites for this factor and determined what kind of additional contextual rules appeared to be molecular descriptors for these sites. We used two machine learning techniques for revealing such rules and applied them to building methods for the recognition of functional genomic HNF4α sites. The techniques used here are similar to the methods applied for the recognition of AhR sites [2]. The main features of the techniques as well as their recent improvements are reported here.

#### Defining local context: revealing short sequence motifs with the help of an exhaustive feature selection algorithm

Initially, we interrogated short sequence motifs with the help of an exhaustive feature selection algorithm and searched for cliques in the feature correlation graph. Specifically, we analyzed flanking regions of HNF4α bindings sites, 28 bp upstream and 33 bp downstream, and applied a modification of the search algorithm that was recently developed [14,33]. It should be noted that the algorithm searches for a specific composition of over- and underrepresented short oligonucleotides (features of local context) in the flanking regions of HNF4α sites and uses them for construction of a site recognition method. In the first step, the algorithm - through an exhaustive search - selects a set of such features of the local context. In the second step, it creates a graph of correlations between the features, selects non-redundant combinations of them through identification of cliques in the graph and builds the site recognition method using a final set of features of local context.

The algorithm compares two sets of sequences of equal length L: a training set Y_Local _consisting of the functional HNF4α sites including their flanking regions (Additional data file 1), and a background set of sequences N_Local_. The N_Local _set is made by running the TRANSFAC^® ^accompanying tool Match™ [55] in the set of human intergenic regions (located at least 1 Mb from any known gene) using the HNF4α PWM (accession number M00967) with score cut-off value *q*_*cut*-*off *_= 0.8. This cut-off guarantees recognition of all known sites collected in Additional data file 1. Then, we randomly selected 100 matches together with their -28 bp and +33 bp flanks and placed them into the background set N_Local_. Therefore, the set N_Local _consists of sequences that contain a central motif fitting to the HNF4α matrix; however, because of its position in the genome so far from any known gene and also since the threshold was so low in the motif matching, a randomly chosen subset is likely to contain mostly false positives. By comparison of the sets Y_Local _and N_Local _we could reveal contextual features that characterize the sequence environment (local context) of functional HNF4α sites. In addition, such comparison allowed us to reveal features of the core motif at the HNF4α binding site that are not captured by the PWM method alone (for example, correlation between positions of the site).

We extended the approach described in Kel *et al*. [2] and consider now three types of contextual features (*φ*). First is the frequency of occurrence of short motifs *λ *= (*a*_1_*a*_2_..*a*_*k*_) (*a* ∈ {*A*, *T*, *G*, *C*, *W*, *S*, *R*, *Y*, *M*, *K*, *B*, *V*, *H*, *D*, *N*}) (we use the following one-letter code for different combinations of alternative nucleotides: W-(A/T (read A or T)); R-(A/G); M-(A/C); K-(T/G); Y-(T/C); S-(G/C); B-(T/G/C); V-(A/G/C); H-(A/T/C); D-(A/T/G); N-(A/T/G/C)) of length *k *≤ *4 *in a window *w *= [*t*_1_,*t*_2_] (*0* <  *t*_2_ <  *t*_1_ <  *L* - *k* + *1*). Second is the frequency in the same window of dinucleotide pairs: (*λ *→ *δ*), where *λ *= (*a*_1_*a*_2_) and *δ *= (*b*_1_*b*_2_) with a distance between them varying from *r*_*min *_to *r*_*max*_. Third is the frequency of four-nucleotide repeats *λ*^2 ^= (*λ *→ *λ*), where *λ *= (*a*_1_*a*_2_*a*_3_*a*_4_) with the varying distance *r*_*min *_to *r*_*max*_.

In our previous work [2,14,33] we described the statistical criteria based on utility theory that permit an identification of single motifs *λ *and the windows *w *that are characterized by a significant difference in their frequencies *f*(*λ*,*w*,*S*) in the sequences S from the sets Y and N. Here, we extend this algorithm to the identification of significant pairs of dinucleotides and four-nucleotide repeats. Found contextual features are then used for creating a context analyzer that is able to perform additional filtering of the potential sites as revealed by the weight matrix method.

The context analyzer is developed in two steps. In the first step, we perform feature selection; we analyze the correlation between all contextual features found by the statistical criteria described above and choose a limited set of features that are characterized by the lowest level of mutual correlations by means of an algorithm revealing maximal cliques in a weighted graph. The contextual features selected at the previous step (*ϕ*_1_, *ϕ*_2_, ..., *ϕ*_*m*_*)* (10-20 relatively independent features) are used for construction of a linear classification function discriminating sets Y_Local _and N_Local_. So, for every sequence *X *we calculate the score of context:

$$d=\beta +\sum_{i=0}^{m}\alpha_{i}\times f(\phi_{i},w_{i},X)$$

where *α*_*i *_and *β *are the coefficients of the discriminating function. These coefficients are obtained through the least-square method of estimating linear regression.

To validate the obtained context analyzer, we applied it to the control sets, Y_Local-Control _and N_Local-Control_, which do not contain sequences used at the training steps. The set Y_Local-Control _consists of new HNF4α sites annotated most recently in TRANSFAC^® ^and N_Local-Control _was constructed through the same procedure as described above but using also randomly chosen but different human intergenic regions compared to those used to construct N_Local_.

It should be mentioned here that repeating the same training procedure with a new random negative set may result in a different set of features for the context analyzer. This happens due to the procedure of feature selection, which does not necessarily select the full set of important features but gives the most discriminative sub-subset of 'representative' mutually uncorrelated features. Different independent runs of the algorithm can result in different subsets of features, although, often, the most discriminative features do not change, and several other features, being literally different, nevertheless represent quite similar oligonucleotides in similar position windows. Such different sets of contextual features usually achieve similar recognition power (similar levels of sensitivity and specificity) and can characterize different 'sub-populations' of the training set of the sites by looking at it from a different 'angle' of different background sequences. Under the conditions of a rather limited training set of real sites, and in order to avoid overfitting, we can not increase the number of selected features too much. So, for further analysis we take the set of mutually uncorrelated features obtained in the first run of the exhaustive feature selection algorithm.

#### Defining the global context: identification of composite modules in HNF4α-targeted promoters using a genetic algorithm

Composite regulatory modules in the promoter regions flanking functional HNF4α binding sites were identified by using the recently developed software tool CMA [56]. Potential TFBSs in the flanking regions were identified by Match™ [55] that uses a library of about 500 PWMs for vertebrate TFs (TRANSFAC^® ^release 9.4).

CMA was applied for analyzing combinations of TFBSs (CMs) in promoters of differentially expressed genes. The definition of a CM is now significantly improved compared to the previous application [2]. It is defined as a set of individual PWMs and pairs of PWMs that are characteristic for co-regulated promoters. CMs are characterized by the following parameters: *K*, the number of individual PWMs in the module; *R*, the number of pairs of PWMs; cut-off value $q_{cut-off}^{\left(k\right)}$; relative impact values *φ*^(*k*)^; maximal number of best matches *κ*^(*k*) ^that were assigned to every weight matrix *k *(*k *= *1*,*K*); cut-off value $q_{cut-off}^{\left(r\right)}$; relative impact values *φ*^(*r*) ^and maximal distances $d_{\max}^{\left(r\right)}$ and maximal number of best matches *κ*^(*r*) ^that were assigned to every matrix pair *r *(*r *= *1*,*R*) in the CM. A CM score is calculated for all sequences *X *according to the following equation:

$$v(X)=\sum_{k=1,K_{i}}\phi^{\left(k\right)}\times \sum_{j=1}^{\kappa^{\left(k\right)}}q_{j}^{\left(k\right)}(x)+\sum_{r=1,R_{i}}\phi^{\left(r\right)}\times \sum_{i=1}^{\kappa^{\left(r\right)}}\left(q_{1,i}^{\left(r\right)}(x)+q_{2,i}^{\left(r\right)}(x)\right)$$

where $q_{j}^{\left(k\right)}(x)$ is the score of the *j-*th match of the *k*-th PWM and $q_{j}^{\left(k\right)}(x)>q_{cut-off}^{\left(k\right)}$; and $q_{1,i}^{\left(r\right)}(x)$ and $q_{2,i}^{\left(r\right)}(x)$ are scores of two sites in a pair *r *and $q_{1,i}^{\left(r\right)}(x),q_{2,i}^{\left(r\right)}(x)>q_{cut-off}^{\left(r\right)}$ and the distance between these sites in the pair: $d_{\min}^{\left(r\right)}<d^{\left(r\right)}<d_{\max}^{\left(r\right)}$. Normalization is then applied as in Waleev *et al*. [15]. So, if *ν*(*X*) is higher than a predefined threshold *ν*_*cut-off*_, the program reports a match of the composite module to the sequence.

The CMA program is based on a genetic algorithm. It takes as input two sets of sequences (the set under study, Y_Global_, and the background set N_Global_) and a set of PWMs for TFs. The program optimizes parameters *K *and *R*, the set of matrices selected, their number, their cut-offs, the relative impact, and the maximum number of best matches. We defined the fitness function of the genetic algorithm as a weighted sum of several statistical parameters characterizing the difference between two distributions - the distributions of the CM scores (*ν*(*X*)) in the two sets of promoters, as described in Kel *et al*. [56]. Calculating the fitness function allows us to assess the usability of the obtained solutions for the classification of individual sequences. The output of the program is the best discriminative CM with the optimized parameters.

### Molecular biology experiments

To confirm predicted functional HNF4α binding sites, we performed electromobility supershift assays with nuclear extracts of Caco-2 cells. Note that this cell line is well characterized for its abundant expression of HNF4α, as reported elsewhere [57].

#### Isolation of nuclear extracts

Nuclear extracts from Caco-2 cells were isolated by a modified method of Dignam *et al*. [58]. Eleven days after seeding, cells were washed twice with ice-cold phosphate buffered saline, scraped into microcentrifuge tubes and centrifuged for 5 minutes at 2,000 × g at 4°C. Cell pellets were resuspended in hypotonic buffer (10 mM Tris, pH 7.4, 2 mM MgCl_2_, 140 mM NaCl, 1 mM DTT, 4 mM Pefabloc, 1% v/v aprotinin, 40 mM β-glycerophosphate, 1 mM sodiumorthovanadate and 0.5% TX100) at 4°C for 10 minutes (300 μl for 1 × 10^7 ^cells), transferred onto one volume of 50% sucrose in hypotonic buffer and centrifuged at 14,000 × g and 4°C for 10 minutes. Nuclei were resuspended in Dignam C buffer (20 mM Hepes, pH 7.9, 25% glycerol, 420 mM NaCl, 1.5 mM MgCl_2_, 0.2 mM EDTA, 1 mM DTT, 4 mM Pefabloc, 1% v/v aprotinin, 40 mM β-glycerophosphate, 1 mM sodiumorthovanadate, 30 μl for 1 × 10^7 ^cells) and gently shaken at 4°C for 30 minutes. Nuclear debris was removed by centrifugation at 14,000 × g at 4°C for 10 minutes. The extracts were aliquoted and stored at -70°C.

#### Electrophoretic mobility shift assay

The oligonucleotides were purchased from MWG Biotech (Ebersberg/Muenchen, Germany); for sequence information see Table 6 (the central five nucleotides are highlighted in bold). Nuclear extract (2.5 μg) and 10^5 ^cpm (0.027 ng) ^32^P-labeled oligonucleotides were incubated in binding buffer consisting of 25 mM Hepes, pH 7.6, 5 mM MgCl_2_, 34 mM KCl, 2 mM DTT, 2 mM Pefabloc, 2% v/v aprotinin, 40 ng of poly (dI-dC)/μl and 100 ng of bovine serum albumin/μl. Oligonucleotides and nuclear proteins were incubated for 20 minutes on ice. Free DNA and DNA-protein complexes were resolved on a 6% polyacrylamide gel. Supershift experiments were done with an HNF4α-specific antibody (sc-6556x, Santa Cruz Biotechnology, Heidelberg, Germany). Gels were blotted to Whatman 3 MM paper, dried under vacuum, exposed to imaging screens for autoradiography and analyzed using a phosphor imaging system (Molecular Imager FX pro plus; Bio-Rad Laboratories GmbH, Muenchen, Germany).

**Table 6:** Shift-probe sequences

Gene symbol*	Gene name^†^	Oligo-name^‡^	Location (rel.TSS)^§^	Score^¶^	Sequence^¥^
*TCF1/HNF1*	*hepatic nuclear factor 1*	HNF1	-265	0.988	AAGGCTGAAGTC**CAAAG**TTCAGTCCCTTC
*APOB*	*Apolipoprotein B*	APOB	-86	0.905	GGAAAGGTC**CAAAG**GGCGCCTTG
*SERPINA1/AAT*	*alpha-1-antitrypsin*	GS21	-134	0.865	CAACAGGGG**CTAAG**TCCACTGGC
*AGT*	*angiotensinogen*	GS47	-429	0.905	TGCAGAGGG**CAGAG**GGCAGGGGA
*APOC3*	*Apolipoprotein C3*	GS104	-93	0.995	GGCGCTGGG**CAAAG**GTCACCT GC
*CYP2D6*	*cytochrome P450, family 2, subfamily D, polypeptide 6*	GS105	-69	0.989	AGCAGAGGG**CAAAG**GCCATCATC
*TF*	*Transferring*	GS106	-76	0.817	ACGGGAGGT**CAAAG**ATTGCGCCC
*ALDH2*	*aldehyde dehydrogenase 2 family (mitochondrial)*	GS107	-332	0.817	CATTGGGGT**CAAAG**GCACACATT
*APOC2*	*apolipoprotein C2*	GS108	-159	0.916	TGTCTAGGC**CAAAG**TCCTGGCCA
*PCK1*	*phosphoenolpyruvate carboxykinase 1 (soluble)*	GS109	-455	0.923	GGTCACAGT**CAAAG**TTCATGGGA
*NCOA2*	*nuclear receptor coactivator 2*	GS110	-485	0.981	ATGGGAGGG**CAAAG**GGCAATGCC
*TFF2*	*trefoil factor 2*	GS111	-495	0.978	AAGATGGGA**CAAAG**GGCATCGTG
*CHEK1*	*CHK1 checkpoint homolog*	GS112	+5	0.976	AGTGGTGGG**CAAAG**GACAGTCCG
*CD63*	*CD63 antigen (melanoma 1 antigen)*	GS113	-182	0.967	CTGCAGGAG**CAAAG**GACAGAAGT
*SH3GL2*	*SH3-domain GRB2-like 2*	GS114	-393	0.964	CGCCAGGCT**CAAAG**GGCAGGAGG
*RND2*	*Rho family GTPase 2*	GS115		0.923	AGGGCAGGT**CAGAG**TTCAAGCGA
*ESRRBL1*	*estrogen-related receptor beta like 1*	GS116	+63	0.91	CAGAACGGA**CAGAG**TCCAGCGTG
*DDB1*	*damage-specific DNA binding protein 1, 127 kDa*	GS117	-295	0.909	GGGGAAGGG**CAAAG**GGCGCGGAA
*NEUROG3*	*neurogenin 3*	GS118	-225	0.896	GATTCCGGA**CAAAG**GGCCGGGGT
*IL6*	*interleukin-6*	GS119	-149	0.889	ACTAGGGGG**AAAAG**TGCAGCTTA
*AZI2*	*5-azacytidine induced 2*	GS120	-217	0.793	GGACCCCCC**AAAAG**GACACTGAG
*CFL2*	*cofilin 2 (muscle)*	GS121	-676	0.792	CGAGGCGAG**AAAAG**CCCCCCGCA
*GPHN*	*gephyrin*	GS122	+733	0.79	GACTGAGAG**GAAAG**GATAGCACA
*C14orf119*	*Chromosome 14 open reading frame 119*	GS123	-610	0.786	CAAGCGGCT**CAAAG**GGGTGAGGA
*PPP1R3C*	*protein phosphatase 1, regulatory (inhibitor) subunit 3C*	GS124	-142	0.772	CGAGACGTG**CAGAG**AGCTATCTG
*AKR1C3*	*aldo-keto reductase family 1, member C3 (3-alpha hydroxysteroid dehydrogenase, type II)*	GS125	-481	0.763	GAAAATGTA**AAAAG**GCAAATATT
*NPAS2*	*neuronal PAS domain protein 2*	GS126	-395	0.759	GAGCCGGCC**CAGAG**GAGAGGCAA
*SAG*	*S-arrestin*	GS127	-106	0.754	CCTGGGAGA**CAGAG**CAAGACTCC
*CLCN3*	*chloride channel 3*	GS128	-377	0.753	AGCGTCACG**CAGAG**TTCGGATCC
*CBX3*	*chromobox homolog 3 (HP1 gamma homolog, Drosophila)*	GS129	-525	0.748	GCGGAAGGC**TAGAG**TCCTGCTAG

*^†^The gene symbol and gene name of the gene where the corresponding HNF4 site was analyzed. ^‡^The internal name of the oligonucleotide used in the study. ^§^Location of the site in the promoter of the gene. The position of the 5' end of the site is given relative to the TSS. ^¶^The score was computed using the HNF4 PWM constructed in the course of this study (Table 2). ^¥^Sequence of the oligonucleotide used in the study. The central part of the oligonucleotide, which corresponds to the most conserved core of the HNF4 motif, is given in bold.

## Abbreviations

ChIP, chromatin immunoprecipitation; CM, composite module; CMA, Composite Module Analyst; DR, direct repeat; EMSA, electrophoretic mobility shift assay; HNF, hepatic nuclear factor; PWM, positional weight matrix; TF, transcription factor; TFBS, transcription factor binding site; TSS, transcription start site.

## Authors' contributions

AK and JB are responsible for the conceptual design of the study. AK developed the genetic algorithms, the selection schemes and analyzed the results. Computational and simulation analysis were done by AK, VM and RZ. Authors VM and RZ helped to code the algorithms. Authors MN and JB are responsible for the molecular biology studies. AK and JB drafted the manuscript and are responsible for its final content. All authors critically evaluated the data. All authors read and approved the final manuscript.

## Additional data files

The following additional data are available. Additional file 1 includes information on TRANSFAC^® ^database annotated HNF4α binding sites. Additional data file 2 is a table listing the 375 sequences that passed our criteria.

## Supplementary Material

Additional data file 1 TRANSFAC^® ^database annotated HNF4α binding sites. Click here for file

Additional data file 2 The 375 sequences that passed our criteria. Click here for file
