# Supplementary material for: Genome wide prediction of HNF4α functional binding sites by the use of local and global sequence context
Source: Genome Biol. 2008 Feb 21;9(2):R36. doi: 10.1186/gb-2008-9-2-r36 (PMC2374721; doi:10.1186/gb-2008-9-2-r36)
Supplement: Additional data file 1 — TRANSFAC® database annotated HNF4α binding sites. [file gb-2008-9-2-r36-S1.doc]

**Additional file 1.** Genomic HNF4α sites annotated in TRANSFAC database.

| **Training (*)** | **Acc^1)^** | **site_id^2)^** | **Seq^3)^** | **From^4)^** | **To^4)^** | **Description^5)^** |
| --- | --- | --- | --- | --- | --- | --- |
| * | R00114 | HS$A1ANTR_01 | (-) TAAGTCCAC | -134 | -98 | AAT (alpha1-antitrypsin) |
| * | R01183 | RAT$OTC_05 | (-) AAAGGCTAT | 76 | 102 | OTC (ornithine transcarbamylase) |
| * | R01183 | RAT$OTC_08 | (-) AACCTCTGA | 161 | 185 | OTC (ornithine transcarbamylase) |
| * | R01457 | HS$TF_08 | (+) GAGGTCAAA | -76 | -48 | Tf (transferring) |
|  | R02657 | HS$APOC3_01 | (-) AAAGGTCAC | -93 | -70 | apoCIII (apolipoprotein CIII) |
|  | R03034 | MOUSE$TTPA_05 | (+) AAGGTTCAT | -155 | -130 | TTR (transthyretin, prealbumin) |
| * | R03035 | MOUSE$TTPA_06 | (+) AAGGGTCAT | -1 | 0 | TTR (transthyretin, prealbumin) |
| * | R03318 | RAT$POMC_14 | (+) GAGGTTAAG | 24 | 55 | POMC (pro-opiomelanocortin) |
| * | R03405 | HIV1$HIV1_20 | (-) CTGACCTTT | -339 | -317 | HIV-1 (human immunodeficiency virus type 1) |
| * | R03881 | RAT$HNF1_02 | (+) GAAGTCCAA | -69 | -48 | HNF-1 (hepatocyte nuclear factor 1) |
|  | R04454 | HS$BGP_02 | (+) TCAGACAAA | -158 | -137 | BGP (biliary glycoprotein) |
| * | R04478 | HS$F8_05 | (-) ATGCCCCTA | -311 | -279 | factor VIII |
|  | R08877 | RAT$FABPI_01 | (-) AAAGTTCAA | -82 | -69 | FABPI (intestinal fatty acid binding protein) |
| * | R08883 | HS$APOC3_02 | (+) TGGGTCCAG | -745 | -725 | apoCIII (apolipoprotein CIII) |
| * | R08885 | HS$GCC_01 | (-) AAAGTTCAC | -50 | -20 | GCC (guanylyl cyclase C) |
| * | R12074 | RAT$PEPCK_23 | (-) AAAGGTCAT | -451 | -440 | PEPCK (phosphoenolpyruvate carboxykinase, cytosolic) |
|  | R13018 | RAT$HMGCS2_01 | (-) AAGGTCTCA | -104 | -92 | Hmgcs2 (3-hydroxy-3-methylglutaryl-Coenzyme A synthase 2) |
| * | R13031 | RAT$NOS2_01 | (+) GGAGGTCAG | -1353 | -1322 | Nos2 (nitric oxide synthase 2) |
| * | R13032 | RAT$LPK_02 | (-) AGAGTCCAG | -147 | -126 | L-PK (liver-type pyruvate kinase) |
| * | R13033 | RAT$TAT_32 | (-) AAAGAGCAG | -3598 | -3579 | TAT (tyrosine amino transferase) |
|  | R13034 | HS$F11_01 | (-) AAAGTCTAA | -375 | -350 | F11 (coagulation factor XI (plasma thromboplastin antecedent)) |
|  | R13035 | RAT$PEPCK_26 | (-) AAAGGTCAT | -451 | -432 | PEPCK (phosphoenolpyruvate carboxykinase, cytosolic) |
| * | R13037 | HS$AKR1C4_01 | (+) AAAGTCCAA | -701 | -684 | AKR1C4 (aldo-keto reductase family 1, member C4) |
|  | R13048 | HS$HNF4A_04 | (+) TGAGTCAAG | -295 | -283 | HNF4A (hepatocyte nuclear factor 4-alpha) |
| * | R13055 | MOUSE$CYP3A16_01 | (+) AAAGTCCAG | -146 | -55 | Cyp3a16 (cytochrome P450, 3a16) |
| * | R13057 | MOUSE$AFEP_17 | (-) AAAGACCAC | -178 | -146 | AFP (alpha-fetoprotein) |
| * | R13059 | HS$F7_01 | (-) CGGGCAAAG | -73 | -47 | F7 (coagulation factor VII (serum prothrombin conversion accelerator)) |
|  | R13060 | CHIPMUNK$HP25_01 | (+) AAAGTCCAA | -67 | -51 | HP-25 (hibernation-specific protein 25) |
|  | R13063 | MESAU$CYP7A_03 | (-) TAAGTCCAT | -210 | -185 | Cyp7A (cytochrome P450, 7a) |
| * | R13065 | RAT$HNF6_02 | (+) AAAGGCCAT | -657 | -633 | HNF6 (Hepatocyte nuclear factor 6) |
|  | R13067 | RAT$CPT1A_02 | (+) AAAGTTCAA | -2900 |  | Cpt1a (carnitine palmitoyltransferase 1) |
| * | R13070 | RAT$CYP7A_06 | (-) TAAGTCCAT | -146 | -134 | CYP7A (cholesterol 7alpha-hydroxylase) |
| * | R13208 | HS$EPO_07 | (-) GTGGCCCCT | -99 | -78 | Epo (erythropoietin) |
| * | R13209 | HS$EPO_08 | (-) AGAGGTCAG | 145 | 158 | Epo (erythropoietin) |
| * | R13273 | RAT$ALDH3_04 | (-) GTGCCCCAT | -260 | -233 | ALDH3 (aldehyde dehydrogenase, class 3) |
| * | R14239 | RAT$OXT_02 | (-) CTTGACCCC | -172 | -148 | Oxt (oxcytocin) |
|  | R14385 | HS$FIX_05 | (-) AAAGTACAA | -40 | -9 | factor IX (antihemophilic factor B, christmas factor) |
|  | R14648 | HS$PAX4_03 | (-) TCTTGCCAC | -1960 | -1939 | PAX4 (paired box gene 4) |
|  | R15791 | RAT$THIOLASEB_01 | (-) AAGGTCTCT | -688 | -664 | 3-ketoacyl-CoA thiolase B (3-KETOACYL-COA THIOLASE B, PEROXISOMAL PRECURSOR) |
|  | R15841 | HS$AGT_02 | (-) AGAGGGCAG | -429 | -386 | AGT (angiotensinogen (serine (or cysteine) proteinase inhibitor, clade A (alpha-1 antiproteinase, antitrypsin), member 8)) |
|  | R15842 | HS$AGT_03 | (-) AAGGTTCCC | -281 | -252 | AGT (angiotensinogen (serine (or cysteine) proteinase inhibitor, clade A (alpha-1 antiproteinase, antitrypsin), member 8)) |
|  | R15845 | HS$ALDH2_01 | (+) GGGGTCAAA | -332 | -307 | ALDH2 (aldehyde dehydrogenase 2 family (mitochondrial)) |
|  | R15846 | RAT$ALDB_04 | (+) AAAGTTCAT | 2146 | 2184 | ALDB (aldolase B) |
|  | R15854 | HS$AMBP_02 | (+) AAAGTCCAA | -2794 | -2770 | AMBP (alpha-1-microglobulin/bikunin precursor) |
|  | R15891 | HS$ANTHIII_03 | (-) AAAGTGTAG | -126 | -100 | AT3 (antithrombin III) |
|  | R15898 | HS$CYP2D6_01 | (+) AAAGGCCAT | -69 | -28 | CYP2D6 (cytochrome P450, family 2, subfamily D, polypeptide 6) |
|  | R15902 | HS$FIX_07 | (+) AAAGGTTAT | 15 | 40 | factor IX (antihemophilic factor B, christmas factor) |
|  | R15904 | HS$FIX_08 | (-) AAAGTACAA | -23 | 6 | factor IX (antihemophilic factor B, christmas factor) |
|  | R15905 | HS$CYP2C9_03 | (+) TGGGTCAAA | -155 | -140 | CYP2C9 (cytochrome P450, family 2, subfamily C, polypeptide 9) |
|  | R15907 | HS$CYP3A4_06 | (+) CATGTGCAA | -11357 | -11331 | CYP3A4 (Cytochrome P450 3A4) |
|  | R15908 | HS$CYP3A4_07 | (-) TAAGTTCAA | -11172 | 11147 | CYP3A4 (Cytochrome P450 3A4) |
|  | R15916 | HS$CYP8B1_01 | (+) AAGGTCCAG | 198 | 227 | CYP8B1 (cytochrome P450, family 8, subfamily B, polypeptide 1) |
|  | R15917 | HS$CYP27A1_01 | (-) AAAGGCCAG | -120 | -98 | CYP27A1 (cytochrome P450, family 27, subfamily A, polypeptide 1) |
|  | R15921 | HS$GK_01 | (+) AAAGGGCAG | -455 | -428 | GK (glycerol kinase) |
|  | R15922 | HS$HO_07 | (+) TAGGACAAC | -1793 | -1764 | HO (heme oxygenase) |
|  | R15923 | HS$MCAD_03 | (-) AAGGTCAAA | -338 | -308 | MCAD (MCAD (medium-chain acyl coenzyme A dehydrogenase)) |
|  | R15925 | HS$MSP_01 | (+) ACAGTGCAG | 16 | 45 | MSP (macrophage stimulating protein) |
|  | R15927 | HS$MSP_02 | (+) AGGGTCCAG | -135 | -105 | MSP (macrophage stimulating protein) |
|  | R15939 | HS$PPARA_02 | (+) AAAGTTCAC | -1498 | -1476 | PPARA (peroxisome proliferator-activated receptor alpha) |
|  | R15941 | HS$SHBG_02 | (+) GGGGTCAAG | -88 | -66 | SHBG (sex hormone-binding globulin) |
| * | s00023 | C4$C4_001 | (-) AAAGGTCAC | -82 | -69 | apolipoprotein CIII |
| * | s00099 | WH$C4_001 | (+) AAGGTTCAT | -151 | -94 | transthyretin, prealbumin |
| * | s00180 | ZIP$C4_009 | (-) AAAGGGCGC | -81 | -52 | apolipoprotein B |
| * | s00182 | WH$C4_002 | (-) GGGGTCAAG | -213 | -147 | apolipoprotein AI |
|  | s00476 | HOM$C4_006 | (-) TAAGTCCAC | -110 | -48 | alpha1-antitrypsin |
| * | s00487 | C4$C2H2_006 | (-) AAAGTCCAC | -71 | -54 | blood coagulation factor X |
|  | s00489 | C4$NFY_001 | (+) AGGGTCCAG | -28 | 32 | macrophage stimulating protein |
| * | s00491 | C4$C4_007 | (-) CCTGGCCAA | -159 | -81 | apolipoprotein C-II |
| * | s00503 | C4$C4_008 | (+) AAAGTTCAC | -277 | -215 | hepatocyte nuclear factor 1 |
|  | s00504 | C4$C4_009 | (-) TAAGTCCAT | -146 | -128 | cholesterol 7alpha-hydroxylase |
|  | s00507 | C4$GATA_002 | (+) AGAGTCCAG | -85 | 47 | Fetoprotein Transcription Factor |
|  | s00639 | HOM$C4_011 | (+) AAAGTCCAA | -698 | -669 | aldo-keto reductase family 1, member C4 |
| * | s00642 | ZIP$C4_013 | (+) AAAGTTCAA | 3920 | 3985 | carnitine palmitoyltransferase 1 |

**^1)^** acceession number of the site in TRANSFAC® database
**^2)^** identifier of the site in TRANSFAC® database

**^3)^** sequence of the site core and the orientation of the motif relative to the transcription direction: (+) in upper strand; (-) in complementary strand.

**^4)^** positions of the site in the promoter relative to the start of transcription.

**^5)^** gene where corresponding site was identified.

* Sites belonging to the training set for the analysis of local context. Sites that are not marked constitute the test set. Selection of sites into the training set was done randomly.
